# The impact of mindfulness practice on physician burnout: A scoping review

**DOI:** 10.3389/fpsyg.2022.956651

**Published:** 2022-09-20

**Authors:** Hani Malik, Carrie Amani Annabi

**Affiliations:** ^1^Department of Family Medicine, Royal College of Surgeons in Ireland, Medical University of Bahrain, Manama, Bahrain; ^2^Graduate School of Healthcare Management, Royal College of Surgeons in Ireland-Dubai, Dubai, United Arab Emirates

**Keywords:** mindfulness practice, physician, burnout, compassion fatigue, moral injury, COVID-19, healthcare leadership, organizational culture

## Abstract

**Background:**

Physician burnout is a growing phenomenon in current health systems worldwide. With the emergence of COVID-19, burnout in healthcare is progressively becoming a serious concern. Increasing emotional exhaustion, depersonalization, and reduced personal accomplishment threaten the effective delivery of healthcare. Compassion fatigue and moral injury are a considerable risk to the doctor-patient relationship. These issues can potentially be mitigated by mindfulness practice, which has shown promising results in reducing burnout, restoring compassion, and preventing moral injury in physicians.

**Methodology:**

A scoping review was conducted to investigate the effects of mindfulness practice on physician burnout. High-ranking journals were targeted to analyze high-quality studies and synthesize common themes in the literature. Studies conducted on current practicing physicians were included. Mindfulness practice of varying forms was the main intervention studied. Gray literature and studies conducted only on allied health personnel were excluded from this review.

**Results:**

31 studies were included in this scoping review. Mindfulness practice decreased emotional exhaustion and depersonalization while improving mood, responses to stress, and vigor. Self-awareness, compassion, and empathy were also increased in study participants. From this review, four themes emerged: innovations in mindfulness practice, mindfulness and positive psychology, the impact of mindfulness on work and patient care, and barriers and facilitators to physician mindfulness practice.

**Conclusion:**

Mindfulness was widely reported to benefit mental health and well-being, but the studies reviewed seemed to adopt a mono focus and omitted key considerations to healthcare leadership, systems-level culture, and practices. Mindfulness practice is a quintessential component of positive psychology and is inherently linked to effective leadership. A mindful and compassionate physician leader will play a crucial role in addressing current practice gaps, prioritizing staff mental health, and providing a supportive platform for innovation.

## Introduction

Stress is a consistent feature of a physician’s average working day. Doctors often engage in technically demanding work that involves important decision-making encased in uncertainty (Shanafelt et al., 2015). Such complex tasks may contribute to a variety of stressful clinical encounters. Martin (2020) suggests that an ideal work situation should cycle between the zones of comfort and stretch. However, when work demands exhaust an individual’s coping mechanisms, there is a risk of moving toward harmful stages of strain. As pressure slopes toward higher levels, physicians’ mental health is affected as they reach levels of burnout, which impacts work performance and quality. Therefore, it is imperative to address burnout and explore pragmatic solutions to mitigate the potential consequences to healthcare.

## Literature review

### Burnout

Burnout is defined as an escalating phenomenon consisting of emotional exhaustion, depersonalization, and reduced professional efficacy (Maslach and Leiter, 2016). Chronic cynicism and growing unchecked interpersonal work stressors will result in diminished effectiveness and feelings of perceived incompetence (Klein et al., 2020). This cascades into fatigue, poor concentration, and shame from the inability to cope with the situation (Martin, 2020). Maslach et al. (2001) suggest that a mismatch in excessive workload will exhaust an individual’s energy and coping resources resulting in potentially irreversible consequences.

In the model of conservation of resources, Hobfoll (1989) describes the psychological stress that arises in response to actual and perceived threats to resources, and how this contributes to what is now known as burnout. The author outlines how expended resources that are unable to offset further loss, can result in loss spirals which amplify stress. Burnout subsequently arises from resource depletion and overload. From the workplace perspective, the Job Demands-Resource (JD-R) model illustrates that when job demands are high, additional effort is required to maintain effective performance and achieve intended work outcomes (Demerouti et al., 2001). Schaufeli and Bakker’s (2004) revised version of JD-R observes the protective effects of engagement, or a positive work-related state of mind characterized by vigor, dedication, and absorption, in working individuals. However, when job demands overwhelm resources, coping and recovery, employees succumb to physical and mental exhaustion, which gradually develops into burnout (Schaufeli and Taris, 2014).

### Physician burnout and geographical diversity

In healthcare, burnout is becoming a global predicament that indiscriminately affects varying specialties. An Irish study conducted on 1,749 doctors revealed that more than one-third suffer from burnout (Hayes et al., 2017). Furthermore, the prevalence of burnout has exceeded 50% in American physicians (West et al., 2016), which coincides with the deterioration of work-life balance (Shanafelt and Noseworthy, 2017). A Dutch cross-sectional study on 958 participants observed 29% of physicians suffering from depression while 24% reported varying anxiety levels (Ruitenburg et al., 2012). Also, Hasan et al.’s (2015) study on 230 doctors revealed that 43.1% of Bahrain’s secondary care physicians displayed high levels of emotional exhaustion while 51.5% admitted to low personal accomplishment. A more recent Bahraini study on 211 participants reported a burnout prevalence of 41.2% in Primary Care Physicians (PCP) (AlUbaidi et al., 2020). Burnout appears to be a common phenomenon requiring further inquiry.

When left unaddressed, physician burnout results in a cascade effect. Job stressors and internal strain impair emotional regulation, further compounding destructive attitudes of increased cynicism (Maslach and Leiter, 2016). An Australian study found that 16% of PCPs were depressed while more than 50% contemplated resignation due to work-related stress (Schattner et al., 2010). Distressed physicians struggle with maintaining interpersonal relationships, resulting in mental health decline and substance abuse (Patel et al., 2019). In addition to the high prevalence of physician burnout in Bahrain, Malik et al. (2017) discovered that more than one-third of PCPs suffer from varying degrees of depression, anxiety, and stress. Bedolla and Andrew (2018) argue that with increasing hopelessness, physician burnout can increase suicide risk. A Japanese study discovered that 5.8% of doctors had suicidal ideation multiple times per week (Wada et al., 2010). This association was also prevalent in France, where Lheureux et al. (2016) observed that emotional exhaustion is associated with a suicidal tendency in a sample of 1890 PCPs.

### Burnout during patient care

When physicians persevere through burnout, there are several implications for patient care. Chronic stress reduces concentration during clinical decision-making and erodes communication skills in the doctor-patient relationship (Irving et al., 2009). As a result, healthcare professionals (HCP) struggle to meet work demands. Physicians are at an increased risk of medication and diagnostic errors, which leads to poor quality care and adverse outcomes (Patel et al., 2019). Shanafelt et al. (2010) discovered that higher levels of burnout and depression in surgeons were strongly linked to increased reported medical errors. Maslach and Leiter (2016) warn that burnout depletes productivity and job satisfaction, diminishing organizational commitment. Physicians’ inability to cope can impact the efficacy of health systems (Lheureux et al., 2016). Inevitably, burnout threatens patient safety and may incur further healthcare costs (Noseworthy et al., 2017). Thus, burnout prevention benefits doctors, their patients, health systems, and entire communities.

### Compassion fatigue and moral injury

Newer terminology is being used to describe the reticent suffering endured by HCPs. Due to the emotional and physical demands of providing healthcare services, clinicians can deplete their empathy and objectivity (Pfifferling and Gilley, 2000). A doctor’s compassion for patient care is a finite resource that can diminish with time (Celia, 2019). The resulting mismatch between stressors and coping results in emotional and spiritual exhaustion, manifesting as compassion fatigue (Jablow, 2017). In a United Kingdom (UK) study on 1651 doctors, McKinley et al. (2020) observed that 26.2% have high secondary stress syndrome while 30.7% have low compassion satisfaction. Campbell (2020) reports that 31% of these UK doctors have high burnout and compassion fatigue levels and often blame themselves for the inability to adapt to daily pressures. Compassion fatigue occurs internally as the physician tries to deal with the secondary stress (Babineau et al., 2019). As part of this continuum, physicians resort to maladaptive coping mechanisms. Self-distraction and self-blame become habits frequently used by overworked doctors, fueling the frustration and anxiety that potentially results in full-scale burnout (McKinley et al., 2020).

**FIGURE 1:**
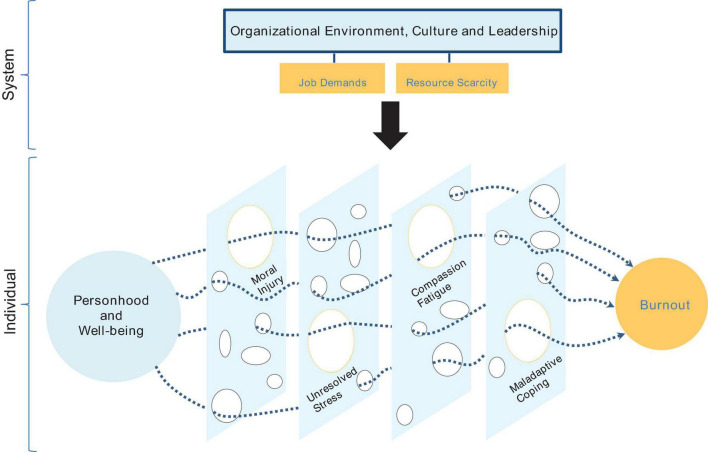
The system individual burnout spectrum model (authors’ own).

Any patient encounter can result in various emotions, often not within a physician’s control, whether life-threatening or routine. Due to the complex doctor–patient relationship, mental health issues like compassion fatigue and burnout in HCPs may develop. Physicians face competing allegiances and thus fail to meet patient needs, resulting in the collapse of resilience (Talbot and Dean, 2018). However, physicians have resisted the burnout classification due to a subtle difference in terminology that may reframe the cause and required intervention (Dean et al., 2019). Talbot and Dean (2019) argue that recurring moral injury plays a significant role in the adversity faced by physicians. Moral injury is defined as “perpetrating, failing to prevent, bearing witness to, or learning about acts that transgress deeply held moral beliefs and expectations” (Talbot and Dean, 2019, p. 3). Recurring moral injury can also occur in tandem with compassion fatigue. Faced with staff shortages and time constraints, UK frontline doctors often see a large volume of patients, and it is unclear if resilience is an effective solution in such circumstances (McKinley et al., 2020). The authors surmised that respondent doctors like emergency physicians and PCPs are more stressed and compassion fatigued compared to other specialties. The System Individual Burnout Spectrum (SIBS) model in Figure 1 illustrates the relationship between the organization and individual, and how job demands and resource scarcity are active stressors on personhood and well-being. As a result, large gaps are formed by moral injury, unresolved stress, compassion fatigue and maladaptive coping, which are catalysts for burnout. However, regardless of disputes over terminology and causality, these issues collectively set a dangerous precedent for healthcare outcomes.

### Mental health during coronavirus disease 2019

In 2019, HCPs in China faced a formidable new adversary, Coronavirus Disease 2019 (COVID-19). By February 2020, 3387 of the 41600 Chinese HCPs employed on the frontlines became infected, accounting for 4% of confirmed COVID-19 cases, while 22 lost their lives (Zhu et al., 2020). The novel coronavirus rapidly infected large populations of several countries, resulting in an unprecedented pandemic.

In addition to China, other countries that COVID-19 significantly impacted include Italy, Spain, and the United States (US). By April 2020, 12000 doctors and nurses in Italy were infected, and 254 HCPs lost their lives (Giusti et al., 2020). It is no question that the COVID-19 poses a significant risk to frontline workers, but several studies have also noted the impact it continues to have on mental health. In a global survey of 2707 HCPs, 51% reported emotional exhaustion, with burnout prevalence recorded in 62.8% of American HCPs (Morgantini et al., 2020). Giusti et al. (2020) reported moderate to severe emotional exhaustion and reduced personal accomplishment in 60% of participants, while 25% were found to have moderate to severe depersonalization. Meanwhile, Luceño-Moreno et al. (2020) observed high levels in Spain, with 41% feeling drained emotionally, while over 50% of HCPs were reported to suffer from post-traumatic stress disorder and anxiety. As the initial epicenter of COVID-19, high levels of burnout were also found in China. Liu et al. (2020) observed that out of 880 participants, over 50% of HCPs had depersonalization and reduced personal accomplishment. Another study on 165 participants observed that 45.6% of doctors and 43% of nurses suffer from symptoms of depression (Zhu et al., 2020). These alarming rates illustrate how the pandemic has put further pressure on an already stressed health workforce struggling to come to terms with this new reality.

COVID-19 has shown that the resulting burnout in overworked HCPs is a universal and collective challenge. In addition to fears of getting infected and spreading it to loved ones, HCPs must deal with an increased workload (Hu et al., 2020). Due to depleting resources, frontline workers were also forced to make difficult decisions in prioritizing which patients receive lifesaving care versus those who would not (Morgantini et al., 2020). Thus, feelings of uncertainty, isolation, and hopelessness became recurring and insidious themes as HCPs struggled with managing a large volume of deteriorating patients (Giusti et al., 2020). Liu et al. (2020) revealed that COVID-19’s high infectivity risk to health staff is directly related to increased emotional exhaustion and depersonalization. Also, Morgantini et al. (2020) discovered that concerns pertaining to scarcity of personal protective equipment (PPE) and restrictions to household activities that HCPs endured were significant principal stressors. Wearing PPE for lengthy periods was uncomfortable, but Hu et al. (2020) reported in their study on 2,014 nurses that 94.8% reported face skin lesions because of PPE use. Furthermore, in Spain, younger HCPs showed higher anxiety and post-traumatic stress levels, possibly due to a lack of work experience coupled with the stress generated from fear of spreading the infection (Luceño-Moreno et al., 2020). Moral injury arises as physicians are forced to balance their safety with the needs of patients and loved ones (Galbraith et al., 2020). Despite nurses suffering from significant burnout, Hu et al. (2020) also report that 96.8% still express commitment to frontline work during this pandemic. This is an encouraging testament to the resolve of HCPs and stresses the need to protect their interests during this challenging time.

The impact of the COVID-19 on physicians is twofold. Mental and bodily strain from working long hours in a risky clinical environment is confounded by the risk of contracting the virus and spreading it to colleagues, patients, and loved ones. As COVID-19 continues exhausting healthcare resources, physician burnout, unfortunately, becomes more predictable.

### Mindfulness practice

From a quality and safety perspective, proactive solutions are needed to address increasing physician burnout. As a countermeasure to evolving mental health concerns, there is growing evidence that practicing mindfulness can positively impact HCP well-being. Epstein (1999) proposed that a mindful practitioner, equipped with clarity and insight, can enhance critical reflection, and effectively manage future challenging situations. As a pioneer in western mindfulness practice, Kabat-Zinn (2003) defines mindfulness as “the awareness that emerges through paying attention, on purpose, and non-judgmentally to the unfolding of experience moment by moment” (p. 145). When practicing mindfulness, thoughts and emotions are acknowledged as passing events that are disconnected from habitual patterns of cognitive and behavioral reactivity (Bishop et al., 2004). A greater sense of autonomy and patience arises within the individual who feels empowered by a non-judgmental acceptance of both pleasant and unpleasant life experiences (Kostanski and Hassed, 2008). Bishop et al. (2004) advocate for mindfulness as a form of mental training where a practitioner observes a thought without distraction. Over time, it can reduce the susceptibility to emotional distress. At its core, such practice creates a genial climate toward thoughts, emotions, and events (Crane et al., 2016). Mindfulness can facilitate new and more effective responses to negative behaviors and ruminating thoughts (Fortney et al., 2013).

Mindfulness is often viewed synonymously with meditation. As experienced during transcendental meditation, mindful practitioners can nurture increased concentration and relaxation (Kabat-Zinn et al., 1992). Subsequently, attention to the present moment and self-awareness are pivotal aspects of mindfulness that can be taught and developed through meditation (Kabat-Zinn, 2003). For example, mindfulness encompasses practices such as sitting meditation or can be incorporated into a daily routine like mindfulness during eating, driving, or even handwashing (Dimidjian and Linehan, 2003). Despite originating from Buddhist traditions, mindfulness is a secular practice, not confined to religious ideology or philosophy.

As an interface between personality and cognition, mindfulness is a skill that one learns through long-term practice that fundamentally changes the way of thinking (Kostanski and Hassed, 2008). Regular practice is required with attention given to the present moment. Michel et al. (2014) explain that this self-regulation of attention requires the individual’s complete focus on immediate, perceptual experiences for the process to be successful. Improving skills of self-observation help in the early detection of problems, instigate a change in thought patterns, and promote a range of coping mechanisms, which ultimately leads to improved psychological function (Baer, 2003). This can potentially serve as a countermeasure to physician burnout.

### Impact of mindfulness on healthcare

The literature observes a trend toward mindfulness being a significant deterrent against physician mental health concerns. In their comprehensive review, Irving et al. (2009) surmised that mindfulness interventions can significantly reduce rumination and encourage reflection. Bedolla and Andrew (2018) suggest that mindfulness is a natural remedy for physician burnout by improving performance, emotional stability, and global cognitive function. Individual mindfulness has the potential to effectively reduce burnout scores (Dunne et al., 2019). From a patient care perspective, physicians who practice mindfulness are more effective listeners and respond with more empathy toward patients in the demanding clinical environment (Luchterhand et al., 2015). Therefore, mindfulness practice appears to be a reasonable coping tool for physicians suffering from burnout and warrants further, focused appraisal.

## Methodology

### Study purpose and aim

A scoping review was conducted to map key concepts and explore the impact of mindfulness practice on physician burnout. This review aims to discover the effects of mindfulness on physician well-being and how the this may shape solutions for the mental health issues observed in physicians worldwide.

The results will provide a greater understanding of mindfulness practice in physicians in the current literature. With the ongoing pandemic still devastating health systems worldwide, it is pertinent to observe whether mindfulness can be a practical intervention to combat the effects of burnout on physician health and patient outcomes.

### Methodological approach

A scoping review was the methodological approach implemented for this study. In their seminal work, Mays et al. (2001) clarify that scoping reviews “aim to map rapidly the key concepts underpinning a research area, especially where an area is complex or has not been reviewed comprehensively before” (p. 194). Arksey and O’Malley’s (2005) original framework provided a stepwise foundation for identifying the research question and relevant studies while conducting these reviews. Scoping reviews help identify gaps in practice and function as precursors for future systematic reviews (Munn et al., 2018). The authors also propose that such studies offer a more systematic alternative to literature reviews in assessing evidence in emerging fields where certain concepts require further clarification.

Levac et al. (2010) recommended prescriptive ways to develop the six steps further while classifying scoping reviews as an iterative process that balances feasibility and comprehensiveness. Peters et al. (2020a) proposed further enhancements in aligning objectives, inclusion criteria, and research questions before searching, selecting, extracting, and analyzing the evidence. The frameworks described by Levac et al. (2010) and Peters et al. (2020a) were used to steer the core elements of this scoping review.

Scoping reviews provide valuable insight to decision-makers and stakeholders about emerging concepts within a field of study and how they are evolving in the literature (Peters et al., 2020a). These reviews offer a more robust alternative to concept analysis and can inevitably be more helpful in incorporating studied interventions into practice (Munn et al., 2018). Common themes from the included studies will be extracted and synthesized for this review. As described by Peters et al. (2020a) in step nine, evidence will be summarized, and conclusions will be made and correlated with the implications to healthcare leadership. This will aid in discovering the effects, limitations, potential challenges, and future research considerations of mindfulness in reducing burnout in a specific studied population group of physicians.

### Research question

Peters et al. (2020a) propose using the Population Concept Context (PCC) mnemonic to help construct a title and relevant inclusion criteria, emphasizing the importance of aligning objectives with the research question. Therefore, current practicing physicians or doctors in any healthcare setting will be the studied population. Mindfulness practice will be the central concept and intervention used within the context of full-time, working physicians regardless of clinical specialty, geography, age, or gender. The research question for this scoping review is as follows: “What impact does mindfulness practice have on the burnout experienced by physicians?”

### Search strategy

The search strategy for this scoping study had a two-phase approach. First, a thorough search was undertaken through multiple databases. After the database search, a manual search was conducted, where relevant journals were individually examined. The rationale behind the manual search was to ensure that no studies were missed from the initial database search. Throughout the search strategy, the focus was on highly ranked journals containing high-quality studies on mindfulness as an intervention for physician burnout. The reference lists of included studies were also screened.

### Inclusion and exclusion criteria

Inclusion criteria help readers understand what is being proposed by the study and guide reviewers in essential decisions on how to steer the scoping review (Peters et al., 2020b). Peters et al. (2020a) suggest that scoping studies can include all types of literature regardless of the quality in methodology. The authors also indicate that given the large volume of literature amassed from the study area, reviewers can impose limits based on what sources will be most appropriate. Thus, a deliberate decision was made to exclude gray literature from the results and focus only on high-quality journals. The initial aim was of collating randomized controlled trials (RCT). However, as the search was iterative, quasi-experimental trials, grouped as non-randomized studies (NRS), were also included in the selection process. The NRS identified from the search included research such as longitudinal cohort studies, non-controlled trials, pilot, or feasibility studies. It was expected that systematic reviews might also be located within the search. These reviews were individually appraised to identify relevant studies that adhere to the inclusion criteria, the studied intervention of mindfulness, and the population of practicing physicians.

To ensure that only high-quality studies were used for this scoping study, the SCImago Journal Rank (SJR) was used, specifically the quartile indicator, to help distinguish between articles based on the quality of journals. Quartiles are used to rank journals from highest (Q1) to lowest (Q4) based on impact factor and index (SCImago, 2021). As suggested by Peters et al. (2020a), limits can be imposed on what sources would be appropriate to use for a scoping review. Thus, Q1 and Q2 journals, which represent the top 25% and 25–50% categories, respectively, were considered for inclusion in this scoping review to identify high-quality research. Studies located in Q3 and Q4 journals were excluded from the search. Studies conducted only on allied health professionals such as nurses, or administrative staff were excluded from the selection process. Also, studies not reported in English were excluded from the results.

### Database search

A thorough search of online databases was conducted for studies that answered the research question. Levac et al. (2010) propose that the strength of a scoping study lies in the comprehensive nature and depth of the literature search in the specific field of interest. The following databases were searched using EBSCOhost: AMED, CINHAL Complete, MEDLINE, APA PsychArticles, APA, and PsychInfo. Google Scholar was also searched for completeness. Multiple databases were examined to ensure a comprehensive approach to the search strategy. Keyword strings were formulated around mindfulness, burnout, and physicians. The search string for mindfulness included: “mindfulness” OR “mindfulness based intervention” OR “mindfulness based stress reduction” OR “meditation.” The search string for burnout included: “burnout” OR “burning out” OR “burntout” OR “burn-out” OR “burn out.” For physicians, it included: “healthcare professional” OR “health professional” OR “doctor” OR “physician” OR “clinician” OR “resident” OR “intern” OR “medical professional.”

Burnout in physicians has been an ongoing, pervasive issue for several years, further amplified recently by COVID-19 and its continuing impact on health systems worldwide. Li et al. (2020) listed a growing body of research on general negative impacts that COVID-19 posed on mental health but they highlighted that this academic literature mostly fell between the two camps of being either commentaries or cross-sectional studies. Linking the expanded requirement for mindfulness to COVID-19, whilst relying on pre-COVID research, is consistent with other laudable work which also acknowledges that the bona fide research prior to 2019 has relevance to the pandemic and post-pandemic era (Tement et al., 2021; Marotta et al., 2022; Sampei et al., 2022). These writings also tacitly acknowledge the paucity of published work in the last 3 years that provide concurrent academic sources. Thus, results were filtered to the last 20 years to keep the literature contemporary, which may help in understanding what has already been researched in this field to instruct current practice. The keyword strings of each concept were then combined with the Boolean operator ‘OR’ and ‘AND’ and then further refined by title and abstract. A total of 587 articles were retrieved from the database search, and after the initial screening with the analytical tool Covidence, 207 duplicates were identified and removed, resulting in 380 studies.

### Manual search

A manual search was conducted through relevant Q1 and Q2 journals to ensure no high-quality studies were missed through the database search. Two guides were used to identify these journals: the Australian Business Deans Council (2019) journal quality list (ABDC) and the Chartered Association of Business Schools (2021) Academic Journal Guide (AJG). Using the ABDC journal quality list, the focus was mainly on journals ranking A*, A, or B. As a result, 32 journals were identified relevant to the study area for this scoping review. Similarly, with the AJG, journals ranking 4*, 4, or 3 were considered, resulting in 24 journals. Additionally, using SJR, 48 highly ranked journals were found. After cross-referencing journals through these three search modalities and eliminating duplicates, 104 high-quality journals were identified for consideration.

An online, manual search was conducted through the 104 journals, with only 14 journals containing relevant studies to this scoping review. While searching these 14 journals, a total of 42 studies were extracted. Within these 42 studies, six studies were duplicates already obtained in the initial database search, and five were irrelevant to the topic. The remaining 31 studies were not considered for the results as they did not meet the inclusion criteria.

### Study screening, data extraction, and charting

With 380 studies extracted from the database search and no includable studies found in the manual search, further screening was conducted to ensure the studies were specifically relevant to the research question and PCC. Levac et al. (2010) recommend that screening abstracts and reviewing full articles is a critical step in refining the search strategy. Data extraction is an iterative process that may require numerous refinements to align objectives with the scoping review’s research question and purpose (Peters et al., 2020a). The authors suggest modifying data charting methods to meet review requirements and to include study details about participants, concept, context, methodology, and critical findings that address the research question. For this scoping review, data was charted in Microsoft Excel and included study authors, SJR quartile indicator, year of publication, study location, intervention type, comparator, duration of study, aims of the study, methodology, outcome measures, and important results Each separate study was charted, continually updated and appraised as part of an iterative process. Commonalities observed in study methodologies, interventions and outcomes formed the basis for theme synthesis.

**FIGURE 2:**
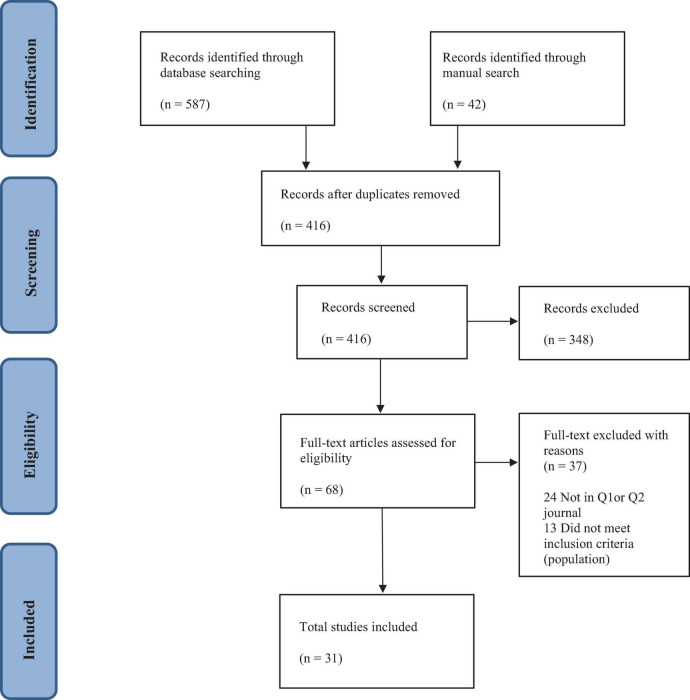
Study selection process.

As an inclusion criterion for this scoping review, high-quality studies on mindfulness and physician burnout in high-ranking journals were targeted, narrowing down the number of studies to 68. Following this step, the studies were screened for eligibility using the SJR’s quartile indicator for Q1 and Q2 categories, which resulted in 44 studies for review. Of the 44 studies, four were excluded as they did not strictly meet the inclusion criteria. In addition, nine studies were systematic reviews that once individually appraised, resulted in only two RCTs to be added to the resulting search. The final total of high-quality studies found in Q1 and Q2 journals included in this scoping review was 31 studies. Results and common themes that emerged from these studies will be discussed and correlated to healthcare leadership, innovation, and organizational culture. The study selection process and search results are summarized in a PRISMA flowchart in Figure 2.

## Results

The scoping review features 31 studies published between 2005 and 2020. Twenty-one studies focused on doctors working in varying specialties as the intervention group. The remaining ten included doctors and a mixed population of HCPs such as nurses, allied health, and support staff. Furthermore, 28 of the 31 studies in this review were published within the last 10 years, with eight publications in 2020. Twenty-two studies were written and conducted in developed, English-speaking countries. This may be attributed to the review’s inclusion criteria or that studies in other languages were not translated and published in English and therefore not discovered during the main search.

The studies in this scoping review examined varying mindfulness interventions that either mitigate physician burnout or facilitate HCP well-being. Eleven of the studies were RCTs in terms of research design, while the remaining 20 were NRS, as shown below in Tables 1, 2. Comparators in the RCTs were mainly active control groups receiving different training content, or waitlist control groups offered mindfulness training after the intervention group completed the program. The number of participants in the studies ranges from seven to 148 health professionals. On average, the RCTs included larger sample sizes than the NRS. However, the duration of mindfulness programs and overall study lengths did not substantially vary between the two categories.

**TABLE 1:** Randomized controlled trials.

Authors and location	Participants	Duration	Intervention	Comparator	Main results
(Shapiro et al., 2005) *US (California)*	38 HCP – mixed group	8 weeks	MBSR	Waitlist control group	Decreased job burnout and stress Increase in quality of life and self-compassion
(Asuero et al., 2014) *Spain*	68 PCPs	29 months	Mindfulness practice, yoga, and group discussions (Krasner’s approach)	Waitlist control group	Decreased burnout and mood disturbance Improved empathy, coping skills and well-being
(West et al., 2014) *US (Minnesota)*	74 Physicians in general medicine	12 months	Physician small-group discussions	Weekly 1 h protected time	Sustained decrease in overall burnout Increased empowerment, work engagement overall quality of life
(Amutio et al., 2014) *Spain*	42 Physicians	12 months	MBSR	Waitlist control group	Increased relaxation levels and reduced heart rate Improved overall energy and coping skills
(Amutio et al., 2015) *Spain*	42 Physicians from multiple specialties	12 months	MBSR	Waitlist control group	Sustained decrease in burnout, heart rate and blood pressure levels Improved mindfulness
(Ireland et al., 2017) *Australia*	44 Intern doctors in A/E	10 weeks	Program based on MBSR, MBCT, ACT	Active control	Significant reduction in stress and burnout Increased resilience
(Verweij et al., 2017) *Netherlands*	148 Physicians from multiple specialties	24 months	MBSR + 1 6 h silent day	Waitlist control group	Improved perceived well-being, personal accomplishment, and self-compassion
(Lebares et al., 2018) *US (California)*	21 Surgical interns	12 months	Modified MBSR + daily home practice	Active control	Participants found concepts and skills useful personally and professional
(Lebares et al., 2019) *US (California)*	21 Year 1 surgical residents	12 months	Modified MBSR + daily home practice	Active control	Improvements in mood, perceived stress, well-being Potential benefits to executive function
(Cascales-Pérez et al., 2020) *Spain*	58 HCP – mixed group	12 months	MBSR	Theoretical training with no practical activities	Increased quality of life, mood, and compassion satisfaction Improvements in mindfulness and burnout
(Ameli et al., 2020) *US (Maryland)*	78 HCP – mixed group	9 months	MBSC	No intervention (life-as-usual)	Reduced stress and anxiety Improved positive affect and mindful selfcare

**TABLE 2:** Non-randomized studies.

Authors and Location	Participants	Duration	Intervention	Main results
Galantino et al. (2005) *US (Pennsylvania)*	84 HCPs – mixed group	8 weeks	Cognitive behavioral stress management course based on MBSR	Decreased burnout levels Significant improvement in mood and vigor
Krasner et al. (2009) *US (New York)*	70 PCPs	15 months	MBSR including group discussion	Improvements in all burnout subscales Sustained improvements in well-being Increased resilience and empathy
Brady et al. (2011) *US (Michigan)*	16 HCPs – mixed group	4 months	Modified shortened MBSR	Decreased stress levels Improved self-care
Beckman et al. (2012) *US (New York)*	20 PCPs	Interviews conducted after program completion	52-h mindful communication program	Improved self-awareness, perceived well-being Enhanced workplace collegiality and reduced professional isolation
Fortney et al. (2013) *US (Wisconsin)*	30 PCPs	9 months	Abbreviated MBSR	Sustained improvements in burnout levels, depression, anxiety, and perceived stress
Pflugeisen et al. (2016) *US (Washington)*	23 Physicians from multiple specialties	16 weeks	Online video-module mindfulness training	Sustained decreases in stress and burnout levels Increased awareness and mindfulness skills
Dobkin et al. (2016) *France*	27 HCPs – mixed group	4 months	MBSR	Reductions in stress and burnout Increases in mindfulness and resilience
Kinser et al. (2016) *US (Virginia)*	49 HCP – mixed group	20 months	Mindfulness curriculum program for Interprofessional HCPs	Reductions in perceived stress, burnout, and anxiety Enhanced personal accomplishment over time
Razzaque and Wood (2016) *UK*	26 Psychiatrists	2 days	Mindfulness-based professional development retreat	Reduced perceived burnout Enhanced emotional regulation and perceived mindfulness
Wen et al., 2017 *US (California)*	50 Resident physicians from multiple specialties	1 month	Mindfulness-based app (Headspace)	Increase in positive affect and improved perceived well-being
Bentley et al. (2018) *US (North Carolina)*	7 Year 1 Psychiatry residents	8 weeks	Mindfulness and empathy training program adapted from Krasner’s approach	Decreased burnout levels Improvement in empathy and reflective listening
van Wietmarschen et al. (2018) *Netherlands*	54 PCPs	6 months	MBSR	Sustained reductions in perceived stress Sustained increase in positive affect Increased physician empathy
Braun et al., 2019 *US (Virginia)*	18 HCP – mixed group	7 months	MIHP (Mindfulness for Interdisciplinary Health Professionals)	Decreased burnout levels Increased dispositional mindfulness and awareness of selfcare
Bu et al. (2019) *UK*	20 Foundation year physicians	3 months	Program adapted from Mindfulness in the Workplace course	Decreased stress levels Improved emotional well-being, working life, and doctor-patient relationship
Janssen et al. (2020) *Netherlands*	30 HCPS – mixed group	26 months	MBSR	Decreased burnout levels Improvements in general mindfulness, positive emotions quality of sleep, and self-efficacy
Minichiello et al. (2020) *US (Washington)*	17 Family physician residents	5 months	Mindfulness-skills based training course	Sustained decreased depersonalization and perceived stress Improvement in mindful awareness and resilience
Roy et al. (2020) *US (Massachusetts)*	57 Physicians from multiple specialties	3 months	App-based mindfulness training program	Sustained decrease in cynicism and emotional exhaustion
Nguyen et al. (2020) *US (Ohio)*	66 Physicians from multiple specialties	4 months	Mind-body skill training	Reductions in burnout and emotional exhaustion Improvement in compassion
Fendel et al. (2020) *Germany*	9 Resident physicians	2 weeks	MBSR	Reduced perceived stress and work-related burnout Improvements in self-compassion, and physician empathy
Hente et al. (2020) *US (Ohio)*	24 HCP – mixed group	15 months	MBCT	Improvements in perceived stress, depersonalization, anxiety, and resilience Sustained improvements in empathy, perspective taking and depressive symptoms

### Early mindfulness interventions

With burnout in HCPs reaching epidemic levels in recent times, it is interesting to note that mindfulness interventions have been studied earlier in the literature. The Mindfulness-Based Stress Reduction (MBSR) program used by Shapiro et al.’s (2005) US RCT on 38 HCPs, reported decreased job burnout and distress. The authors also found that 88% of HCPs had improved stress scores, and 90% reported increased self-compassion. Furthermore, Galantino et al.’s (2005) US study on a mixed group of HCPs reported decreasing emotional exhaustion and depersonalization, significantly improving participant mood and vigor. Meanwhile, Krasner et al.’s (2009) seminal study on 70 American PCPs reported improved scores across all burnout subscales. The authors reported that mindfulness moderately correlating with reductions in tension, fatigue, and depression.

### Randomized controlled trials and physician mental health

With RCTs in this scoping review, there are encouraging results of mindfulness being a valuable tool for physician burnout. In the US, West et al.’s (2014) study on 74 physicians observed a decrease in rates of burnout, specifically within the depersonalization and emotional exhaustion components. In contrast, the authors reported increases in depersonalization in the control group, while it dropped by 15.5% in the intervention group and was sustained 12 months after study completion. Another study in the US on 21 surgical residents found statistically significant improvements in mood and perceived stress, with activation of brain regions relating to emotional regulation (Lebares et al., 2019). In contrast, the authors observed a twofold increase in stress and depression in the control group. In a more recent study using the Mindfulness-Based Self-Care (MBSC) approach on a mixed cohort of 78 HCPs, Ameli et al. (2020) also observed reduced post-intervention levels of stress, anxiety, and depersonalization compared to baseline, which was maintained at the 13-week follow-up.

A study in Spain on 68 PCPs found a slight, six-point reduction in emotional exhaustion and depersonalization subscales and an increase in personal accomplishment (Asuero et al., 2014). The authors also reported significant decreases in mood disturbances such as depression, fatigue, and anger in the intervention group, with improvements in participants’ attitudes. Another Spanish MBSR study on 42 physicians also observed a significant reduction in emotional exhaustion (Amutio et al., 2015). The authors also report improved blood pressure and heart rate in the intervention group. These effects were maintained 10 months later in the study’s second phase (Amutio et al., 2015). In a more recent study in Spain, Cascales-Pérez et al. (2020) found significant emotional exhaustion and depersonalization decreases in a mixed cohort of 58 HCPs. The authors also describe a marked reduction in negative emotions such as depression, anger, and fatigue, while vigor showed significant improvement.

### Sustained effects on burnout in non-randomized studies

Despite no comparator groups in the methodology, the NRS in this scoping review provide interesting observations about mindfulness training in physicians. In a US study on 30 PCPs, Fortney et al. (2013) found a significant increase in personal accomplishment and decreased emotional exhaustion and depersonalization following an abbreviated MBSR program. Despite the short, limited exposure to mindfulness training, the authors found the effects were maintained on follow-up after 9 months.

In another US study on a mixed group of 18 HCPs, Braun et al. (2019) discovered significant and sustainable reductions in emotional exhaustion and depersonalization. The authors interviewed the participants and found that mindfulness helped foster improved connections between participants and their patients, which could have contributed to reducing burnout. In a mindfulness curriculum for interprofessional HCPs, Kinser et al. (2016) reported similar declines in depersonalization and emotional exhaustion with significant reductions in perceived stress and anxiety. Wen et al. (2017) discovered significant increases in positive affect scores on 50 physician residents from multiple specialties in the US. These sustained effects after follow-up were a trend found in several other studies in this scoping review (Pflugeisen et al., 2016; van Wietmarschen et al., 2018; Minichiello et al., 2020). Continued effects were also seen in Roy et al.’s (2020) US study, where half of the 57 physicians reported reduced cynicism, and 20% had reduced emotional exhaustion, which was maintained 3 months post-intervention. More recently, Hente et al. (2020) found significant positive effects at the 15-month mark in a study on 23 HCPs that included physicians. The authors observed reductions in anxiety, perceived stress, and depressive symptoms with enhancements in perspective-taking and resilience. Additionally, Minichiello et al.’s (2020) US study on 17 family physician residents helped move participants out of high levels of burnout range while increasing feelings of personal accomplishment, resilience, and mindful awareness. An increase in personal accomplishment was also found in Janssen et al.’s (2020) study on 30 Dutch HCPs, where sleep quality and self-efficacy were also improved.

### Physician well-being in non-randomized studies

The NRS displayed how mindfulness programs contributed to improving physician well-being, communication, and stress management. A mindfulness communication program in the US designed for 20 PCPs, helped participants improve their sense of well-being, deal with distress and compassion fatigue, and offered a convenient way to discuss experiences and emotional reactions (Beckman et al., 2012). A study in the UK on 28 psychiatrists found significantly enhanced emotional regulation (Razzaque and Wood, 2016). The authors report on how the participants also realized the importance of taking a break from work and improving their awareness of working alliances. van Wietmarschen et al.’s (2018) Dutch study on 54 PCPs also found increased participant awareness of feelings and behaviors, facilitating improved perceptions and reactions to stressful thoughts. Similarly, a UK study on 20 foundation year doctors observed reductions in self-reported stress and improvements in responding to stressful encounters (Bu et al., 2019). These results display an important influence of mindfulness on positive psychology.

### Counterarguments and emerging themes

Despite the encouraging results in this scoping review, some studies either did not show improved outcomes or resulted in counterproductive effects. Brady et al.’s (2011) US study on 16 HCPs did not find any significant decrease in emotional exhaustion or depersonalization, while improvements in personal accomplishment were not statistically significant. Similarly, a large MBSR Dutch study conducted over 2 years found no significant difference in depersonalization or emotional exhaustion in 148 physicians from varying specialties (Verweij et al., 2017). However, the authors reported improvements in personal accomplishment, worry, and self-compassion. In addition, such mindfulness courses may increase physician self-awareness and empathy with patients. Still, the seven psychiatry residents in Bentley et al.’s (2018) study reported a reduction in self-reported levels of personal accomplishment.

Disaggregating the NRS and RCTs revealed some variation in terms of total study participants but did not demonstrate any other significant differences. There was no great distinction between studies published in different countries. Therefore, the RCTs and NRS can be viewed as a homogenous entity. The overarching consensus from these studies is that mindfulness may potentially impact physicians and HCPs, regardless of specialty. Several themes also emerged from synthesizing the results of the RCTs and NRS. Four themes were identified: Innovations in Mindfulness Practice, Mindfulness and Positive Psychology, The Impact of Mindfulness on Work and Patient Care, and Barriers and Facilitators to Physician Mindfulness Practice.

## Themes

### Innovations in mindfulness interventions

Structured mindfulness practice appears in several forms. A commonly used method is the MBSR technique. Developed by Jon Kabat-Zinn in 1979, MBSR was initially used to treat psychological morbidity associated with chronic illness in an outpatient clinic setting (Bishop et al., 2004). The course is described as “a training vehicle for the relief of suffering” (Kabat-Zinn, 2003, p. 148) and has also inspired the creation of various other intervention methods. Programs like Mindfulness-Based Cognitive Therapy (MBCT) have shown promising results in treating recurrent major depression (Bishop et al., 2004). Multifaceted approaches like dialectical behavioral therapy use mindfulness to help individuals accept life situations while changing behaviors and emotions (Bishop et al., 2004). In the context of HCPs, the studies in this scoping review showcased several mindfulness innovations.

The MBSR program has sparked offshoots that can be more suitable for HCPs. Fortney et al. (2013) adapted an abbreviated version of the MBSR, which maintained long-term effects for the participating physicians while also being time-efficient and cost-effective. Similarly, Brady et al.’s (2011) 4-week program reduced in-class time to 1 h per week. An advantage of using MBSR in this shorter form is that it optimizes resources toward an improved quality of patient care (Amutio et al., 2015). Continuing the trend of shorter programs, Ameli et al.’s (2020) implemented the 7.5-h MBSC program, which proved to be a practical approach to mindfulness training for busy HCPs. Meanwhile, Hente et al. (2020) used an MBCT program, which resulted in significant reductions in work-related stress in a mixed group of HCPs.

Conversely, Ireland et al. (2017) took a more diverse approach by combining aspects of various mindfulness programs such as MBSR, MBCT, and Acceptance and Commitment Therapy to develop an exclusive intervention for their study. The authors found this unique combination to be flexible, portable, and largely self-directed, which is appropriate for busy physicians. The variety and adjustability of such available programs could be helpful for doctors who may find it challenging to commit to extensive mindfulness training.

There are other ways in which innovative mindfulness interventions can benefit physicians directly at the workplace. Evident in the study by Braun et al. (2019), the implementation of Mindfulness for Interdisciplinary Healthcare Professionals (MIHP) provided a structured course that facilitated healthy dialogue between various specialties. There is value in discussing and relating personal experiences with colleagues within a mindfulness context. The mindfulness-based professional development retreat allowed physicians to cultivate supportive networks and share their passion for mindfulness with each other (Razzaque and Wood, 2016). Such experiential learning can facilitate doctors to immerse themselves into mindfulness practice further, thus creating a positive feedback loop. West et al. (2014) discovered similar benefits with small-group mindfulness discussions, where sharing burnout experiences had a long-lasting, therapeutic effect on participants. This outcome was also evident in Beckman et al.’s (2012) study using a 52-h mindful communication course. For 15 of 20 physicians, sharing personal experiences with colleagues was identified as one of the most meaningful aspects of the course. The authors found that peer-to-peer interactions brought greater awareness to the importance of workplace interactions, solidified collegiality, and reduced the sense of isolation for many physicians in the study.

Due to the portability of mindfulness practice, it is not surprising that technology has innovatively exploited this aspect in several advantageous ways. Nguyen et al.’s (2020) mind-body skill training was delivered as an online module supported by free, downloadable audio recordings for the 66 physician participants. The convenience and privacy of accessing mindfulness training at one’s own pace provide a potential solution to the issue of busy working hours (Nguyen et al., 2020). Pflugeisen et al. (2016) observed similar results where their study on a flexible, audio-visual mindfulness program effectively provides mindfulness training to busy physicians. Smartphone app-based mindfulness programs have also become more prevalent. Because of their low cost and wide availability, app-based mindfulness interventions have become increasingly popular for behavioral treatments (Roy et al., 2020). The use of the app Headspace, as evident in the study by Wen et al. (2017), proved to be a feasible, short mindfulness intervention that improves physician well-being. Furthermore, Roy et al. (2020) developed their own mindfulness training smartphone application with 30 didactic, experience-based modules available to 57 physicians who can access mindfulness exercises in their free time. The access and flexibility of such timely innovations can be helpful for physicians with demanding work schedules.

### Mindfulness and positive psychology

This scoping review explored how mindfulness affects physician burnout. Themes of self-care and increased well-being, in association with mindfulness, emerged. In Ameli et al.’s (2020) study, the MBSC group demonstrated improved positive affect and self-care, while 32 out of 34 study participants reported that the program enhanced their overall quality of life. Evident in the study by Braun et al. (2019), awareness of self-care supported several participants in a perspective shift toward greater self-compassion. Several other studies observed improvements in perceived emotional well-being in mindfulness intervention groups (Verweij et al., 2017; Wen et al., 2017; Bu et al., 2019; Lebares et al., 2019; Fendel et al., 2020; Hente et al., 2020). MBSR intervention group participants in the study by Cascales-Pérez et al. (2020) described a significant increase in positive emotions such as vigor and friendship and reduction in negative emotions.

In addition to increasing self-awareness and compassion, mindfulness practice has reinforced participants’ resolve, empathy, and coping skills. Ireland et al. (2017) found their mindfulness intervention to be potentially effective in increasing resilience in physicians. Furthermore, Krasner et al.’s (2009) use of mindful communication in PCPs lowered their reactivity to stress and strengthened their resilience toward adversity. This was also concluded in the MBSR study by Dobkin et al. (2016), where physicians may be able to improve their resilience and not succumb to compassion fatigue or burnout. In the study by Bentley et al. (2018), participants felt empowered by the self-awareness facilitated by mindfulness, where they felt comfortable with being imperfect and displaying increased empathy. This increase in empathy and being less judgmental of others was also reported in two other studies (Krasner et al., 2009; van Wietmarschen et al., 2018).

The benefits of mindfulness create a positive feedback loop, where feelings of increased well-being promote further practice (Braun et al., 2019). Micro-practices such as meditating while sitting down allowed HCPs to reset during stressful times (Ameli et al., 2020). Several studies reported on doctors incorporating mindfulness into their lives to cope and relax. For example, in the study by Bu et al. (2019), physicians admitted using mindfulness to attain relaxation to help them sleep. Along with surges in positive energy such as optimism and confidence, participants in the RCT by Amutio et al. (2014) obtained increases in basic relaxation levels. In addition, more than 87% of participants in the study by Asuero et al. (2014) reported feeling an increase in overall energy and improvements in coping skills such as effective communication, time management, and better handling of stress. Therefore, mindfulness may synergistically promote positive psychology and has the potential to facilitate an increase in general well-being and quality of life.

### The impact of mindfulness on work and patient care

Competent physicians aim to nurture the doctor-patient relationship as they provide holistic care resulting in favorable outcomes for patients. In the study by Beckman et al. (2012), 12 of the 20 PCPs felt that openness and increased self-awareness enhanced their patient-doctor interactions. As a result, the mindfulness communication program reinvigorated their clinical practices. West et al. (2014) discovered that physicians undergoing mindfulness intervention felt more empowered and engaged at work, and this effect was sustained 12 months after study completion. By developing an awareness and understanding of a patient’s illness experience, physicians can regulate their emotions, lower their reactivity, and have flexibility in their responses during the clinical encounter (Razzaque and Wood, 2016).

Interestingly, the study by Dobkin et al. (2016) revealed that HCPs who completed the MBSR program developed a more mutually understanding relationship with their patients and encouraged open discussion about critical psychosocial issues. More recent studies on physicians have shown mindfulness to have a substantial impact on doctor-patient interactions. van Wietmarschen et al. (2018) report on PCPs who underwent the MBSR program feeling better equipped, focused, and open to varying approaches when dealing with future, complex patients. With MIHP, several participants in Braun et al.’s (2019) study reflected how mindfulness could help HCPs be genuinely present with their clients. Foundation year physicians admitted to being more mindful, patient, and compassionate with challenging patient scenarios after undergoing the mindfulness in the workplace course (Bu et al., 2019). Cascales-Pérez et al. (2020) suggest that by being more attentive to patients, there will be an increase in the quality of working life, which could synergistically further a physician’s capacity for self-compassion and attention. When mastering this technique during a consultation, the patient can reap the rewards, and the physician will have met their management goals.

For effective healthcare delivery, physicians must be able to see the medical encounter from the patient’s perspective. Healthy empathy is vital for HCPs to maintain a balance where they are open to seeing things from others’ viewpoints but do not lose themselves in the experience (Bentley et al., 2018). Awareness is the key to achieving such empathy so that physicians can reappraise complex events and incorporate curiosity and openness into the clinical encounter (Dobkin et al., 2016). This approach can also help avoid compassion fatigue. Bentley et al. (2018) suggest that in addition to reducing stress and burnout, mindfulness training can increase physician empathy. One way this can be achieved is through reflective listening. The psychiatry residents undergoing mindfulness training in the study by Bentley et al. (2018) emphasized the importance of reflective listening while consulting patients and how it helps promote empathy. The authors report on participants describing increases in awareness of cognitive experiences that would help manage both personal and work stressors. In turn, mindful physicians will be better equipped to handle themselves and their patients during challenging clinical encounters.

### Barriers and facilitators to physician mindfulness practice

Due to a physician’s typical work schedule, time constraints can be a significant hurdle in committing to personal obligations. Often, such instances come at the expense of self-care. Shapiro et al. (2005) reported lack of time and increasing obligations as the main reasons participants dropped out of the MBSR program. Furthermore, Galantino et al. (2005) suggest that various other commitments in the HCPs’ lives may have affected study outcomes. Participants in the study by Beckman et al. (2012) cited perceived work and home responsibilities as the most common factor in missing sessions of the mindfulness communication program. In addition to lack of time, unpredictable work schedules and family commitments were significant barriers to practicing mindfulness (Brady et al., 2011; Wen et al., 2017; Bentley et al., 2018; Braun et al., 2019; Bu et al., 2019; Nguyen et al., 2020). Unfortunately, with increasing work demand and stress, physicians may be reluctant to commit to extended mindfulness programs, which may not guarantee an immediate benefit (Nguyen et al., 2020). Therefore, to effectively implement a mindfulness intervention, time issues must be tackled.

One way to work around the lack of time is by adjusting the mode of mindfulness training delivery. Evident in the study by Pflugeisen et al. (2016), the video-module approach requires minimal time commitments as it delivers mindfulness training in a flexible, cost-effective manner. Wen et al. (2017) advocate for smartphone-based apps as they are timely and easy to use. Meanwhile, Ameli et al. (2020) argue that shorter mindfulness programs effectively address attrition due to busy physician schedules. However, mindfulness training needs to be adjusted for the intended group. Tailored programs that take specific work stressors, character traits, and maladaptive coping mechanisms into account may be more effective in improving well-being (Fendel et al., 2020). Furthermore, several studies have also found informal mindfulness practices outside of the structured curriculum to be of value to participants (Lebares et al., 2018; Braun et al., 2019; Nguyen et al., 2020). For example, the “stop-breathe-be” method in the study by Braun et al. (2019) was highly endorsed by participants, as it can be practiced anywhere while also consuming a short amount of time in a given moment. The authors also observed that informal practices could facilitate integrating mindfulness into daily life. Therefore, technology-based, abbreviated mindfulness training fortified by continuous, informal practice can potentially address the issues HCPs have with time.

This scoping review investigated the impact of mindfulness on the burnout experienced by physicians. Mindfulness practice highlights the importance of self-care and self-compassion and improves resilience, coping skills, and perceived emotional well-being. Additionally, mindful communication increases physician empathy and improves working environments, resulting in a more effective doctor-patient relationship. Despite the encouraging facilitators to mindfulness practice in physicians, a considerable barrier takes precedence. Participants in Beckman et al.’s (2012) study noted that mindfulness programs focusing on awareness, well-being, and self-development still fail to address systemic practices. The authors indicate that accommodating mindfulness training in healthcare work environments requires a systems-level change that nurtures effective communication and manages professional isolation. Healthcare leadership will play a pivotal role as a catalyst for such change and will be an essential ally in the physicians’ struggle with burnout.

## Discussion

COVID-19 continues to pose challenges to healthcare worldwide. Health systems are struggling to allocate resources to combat evolving situations. More insidiously, COVID-19 is taking a toll on the mental health of frontline HCPs. With mindfulness, physicians may have a tool that can help alleviate such concerns and improve overall well-being. The research question for this scoping review asked: “What impact does mindfulness practice have on the burnout experienced by physicians?” Eight of the included studies were published in 2020, many coinciding with the peak of the pandemic. Increased interest in mindfulness research on HCPs may be a product of the struggles faced by doctors, which are being amplified by current health urgencies. However, before inferences can be made, the implications of mindfulness and burnout to individuals, existing health systems, and healthcare leadership require an in-depth examination. This section will consider and analyze various other determinants related to burnout in physicians.

### Positive psychology and oxytocin

One of the themes in this scoping review illustrated how mindfulness produces positive emotions in physicians. Mindfulness is a fundamental component of positive psychology (Annabi, 2021). Carr (2020) proposes methods to achieve happiness, two of which are through mindfulness meditation and finding a ‘flow state.’ The world’s leading researcher in positive psychology, Mihaly Csikszentmihalyi (1998), described how individuals completely immerse themselves in the present activity while in the flow state. Thoughts or emotions unrelated to the activity become irrelevant as the focus is maintained on the present moment. Flow experiences allow for efficient and effortless execution of actions (Ito et al., 2019). By focusing on the immediate task, individuals entering a flow state find it enjoyable and intrinsically rewarding (Csikszentmihalyi, 1998).

A flow state is not limited to an individual but can also be experienced mutually in a group setting. In their mixed design study on four jazz vocalists, Keeler et al. (2015) found that group singing reduces stress and arousal while inducing social flow states in pre-composed and improvised musical performances. The authors discovered increased plasma oxytocin levels, specifically in response to impromptu singing. Oxytocin, the hormone intrinsically linked to emotional and social well-being, is also increased through mindfulness meditation (Donadon et al., 2018; Ito et al., 2019). This, in turn, gives the mindful individual a sense of well-being and contentment.

The World Health Organization (WHO) considers mental health as “the foundation for well-being and effective functioning for an individual and for a community” (World Health Organization [WHO], 2004, p. 10). Since flow states result in increased creativity, efficacy, and subjective well-being (Csikszentmihalyi, 1998), achieving flow can enhance mental health. By focusing on the present moment through mindfulness practice, individuals can potentially forge a link with a flow state, resulting in increasing levels of oxytocin, positive emotional states, and overall feelings of well-being.

### Mindfulness interventions in current health systems

**FIGURE 3:**
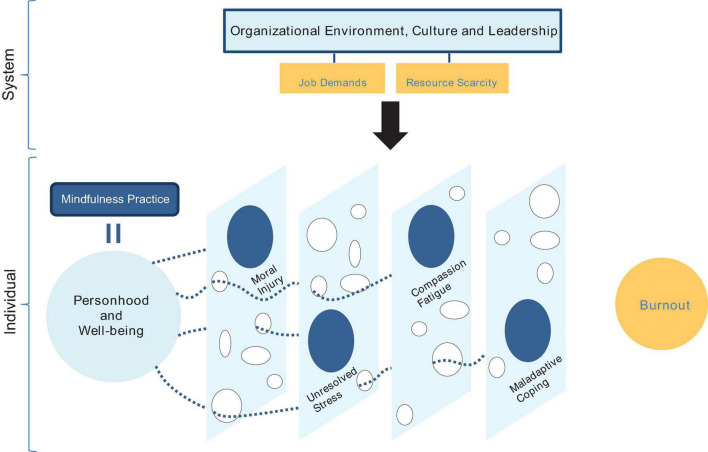
The SIBS mindfulness practice model (authors’ own).

The studies in this scoping review observed homogenous results with minor variations. Mindfulness was linked to increased perceived well-being, potentially reducing physician burnout. As the SIBS Mindfulness Practice model in Figure 3 illustrates, mindfulness practice can block the gaps formed by moral injury, unresolved stress, compassion fatigue, and maladaptive coping. Yet, authors in several studies indicated how physicians struggling with job pressures tend to neglect self-care, which challenges the implementation of any mindfulness intervention. HCPs are not fundamentally susceptible to mental health deficiencies, but mindfulness can pose an added stressor to an already time-pressured group of physicians. However, the most significant finding of this scoping review is that the studies failed to challenge the existing health systems that propagate such pressure on doctors. The methodologies and results of the studies did not incorporate nor address systems-level causality or the baseline for the input of mindfulness training. Increasing patient-related demands, scarce resources, and chaotic work schedules are essentially the product of ineffective health systems. Such environments create conditions that diminish empathy and dissolve coping mechanisms, triggering compassion fatigue, moral injury, and burnout in physicians. In response, mindfulness practice may be a valuable treatment opportunity, but the definitive remedy is ingrained in systems-level practices. Despite the benefits of mindfulness practice shown in Figure 3, plugging the holes will only treat the symptoms but not the cause. If health systems are flawed or dysfunctional, individuals will always be compromised.

In current health systems, physicians face working conditions that create multifaceted dilemmas. The dichotomy of accepting the limitations of the *status quo* while aspiring to provide evidence-based, patient-centered care may impact mental health as described in this scoping review or result in more subtle changes in beliefs through coercive persuasion. In his interview with Diane Coutu, psychologist Edgard Schein, an expert on organizational development, explains that “coercive persuasion is when people are in situations from which they cannot physically escape and are pressured into adopting new beliefs” (Coutu, 2002, p. 102). In healthcare, physicians may not be physically bound as Schein described, but obligations to patient care will compel them to persevere in ineffective environments. Regardless of their resilience or integrity, physicians learn to accept that the system cannot change, perpetuating ineffective practices and stifling creativity and innovation. Any attempts to modify systemic processes are met with resistance, and groupthink takes over the collective mindset and organizational culture. However, the cognitive dissonance may push some doctors toward burnout, which can evolve into depression and anxiety. An alternative yet unfortunate scenario can also unfold where innovative physicians that reject coercive persuasion become pariahs. As a countermeasure, effective leaders are needed to inspire a fundamental systems-level change.

### Implications to leadership in healthcare

Physician leaders are pivotal in the delivery of effective healthcare services. There is increasing evidence that encouraging physicians to take up leadership roles results in positive outcomes for patient care and enhances overall team performance (Hargett et al., 2017). The National Health Service [NHS] Leadership Academy (2010) proposes that physicians demonstrate leadership by developing self-awareness and managing themselves while also continuing their development. The NHS clinical leadership framework also highlights the importance of identifying strengths, limitations, and the effects of stress on one’s behavior and decision-making. Fulfilling work commitments is expected of competent clinical leaders but should not be at the expense of their health (National Health Service [NHS] Leadership Academy, 2010).

To enhance leadership skills, physicians should practice critical reflection. Through reflective deliberating, doctors will be able to consider various motives and influences as they gain multiple perspectives that help in the decision-making process (Wilson and Cunningham, 2013). Porter et al. (2018) endorse the use of action learning techniques where emerging physician leaders critically reflect on experiences at work, which can offset the lack of formal leadership education. In addition, professional resilience is reinforced as physicians gain insight and move toward improved work practices (Wilson and Cunningham, 2013). Mortari (2015) suggests that: “critical reflection is an exercise of liberty” (p. 4) as it promotes self-awareness and empowerment during lived experiences. This is vital for effective leaders and is fortuitously enhanced by mindfulness.

Despite various leadership skills that can be developed, physicians will be limited by the *status quo*. Furthermore, doctors may struggle to apply essential leadership competencies and risk failing to progress in their work or discipline. Beckman et al. (2012) propose a need for a systemic change that supports transparent communication between staff and patients to reduce professional isolation. A team approach strengthened by trust, honesty, and communication is essential for helping physician leaders achieve intended outcomes (Porter et al., 2018). However, health systems must first establish a conducive working environment that views staff mental and physical health as non-negotiable priorities. To achieve this, healthcare needs physician leaders that are compassionate, innovative, and mindful as they instigate a systems-level change.

### Compassionate leadership and innovation

Physicians enduring mental health problems need a leader who is a supportive advocate and a source of inspiration. Whether dealing with staff or patients, effective leaders “empathize and take into account the needs and feelings of others” (National Health Service [NHS] Leadership Academy, 2010, p. 24). However, with growing responsibilities and work stressors, empathy in physician leaders can deplete over time. Therefore, leaders need compassion to proactively contribute to the well-being of others (Hougaard et al., 2018).

In their seminal work on compassionate leadership, West et al. (2017) propose that leaders explore the challenges faced by staff and take effective action to help followers overcome adversity. The authors also believe that such leaders acknowledge the damaging effects of work overload on health and will be able to foster an inclusive and non-threatening environment based on flexibility, teamwork, and improved practices. Compassion increases trust, enhances loyalty, and creates more significant connections between individuals (Hougaard et al., 2020). These characteristics provide the crucial steps toward recovery in physicians with burnout. Moreover, compassionate leadership transcends organizational hierarchy as it enhances intrinsic motivations, altruistic habits, and creative thinking in followers toward the goal of innovation (West et al., 2017).

The National Health Service [NHS] Leadership Academy (2010) instructs physicians to question the *status quo* as they become role models for innovation and promote creative solutions that transform health services. Performance and desired behaviors are enhanced in organizations that align positive, innovative cultures with their vision and strategy (Hisrich and Kearney, 2014). As health systems are being challenged with costs and advances in technology, a compassionate approach to leadership will help support creative thinking and behaviors (West et al., 2017). As a result, such leaders will support HCPs and be more receptive to solutions such as mindfulness training and determined enough to trial such innovations. The literature boasts significant evidence that a compassionate approach to staff challenges improves clinical productivity and patient care (West et al., 2017). When creative thinking and habits become integral to an organization’s culture and strategy (Hisrich and Kearney, 2014), leaders can promote staff inclusivity, which enables collective compassion toward others and further innovation (West et al., 2017).

### The mindful leader

Effective leaders facilitate healthier interactions and improved employee performance (Annabi, 2021). Despite their resilience, compassionate leaders may also succumb to increasing pressures. As a coping response, leaders may become less empathetic to others (Hougaard et al., 2018). Thus, Hougaard et al. (2020) argue that for an effective leadership approach, compassion must also include wisdom, which translates to leadership competence and effectiveness. The authors believe that competence can be enhanced by practicing candid transparency and engaging in daily assertive interactions with others. Leaders must strive toward effective organizational decisions while maintaining a genuine concern for the collective well-being of others. Moreover, one of the ways to cultivate wise compassion is through a regular mindfulness routine (Hougaard et al., 2020).

Mindfulness practice is quintessential in optimizing leadership quality (Lange et al., 2018). As the creator of authentic leadership theory, George (2012) believes that daily mindfulness meditation helps leaders stay grounded and genuine. By facilitating increased self-awareness and presence, mindfulness empowers leaders to be more comfortable with uncomfortable circumstances (Annabi, 2021). Mindful leaders will be able to nurture a passion for their work and enable others to be more effective (George, 2012). Furthermore, mindfulness can increase resilience and counteract the effects of stress and burnout (Kaplan et al., 2017), helping leaders during challenging situations.

Developing compassion is vital for any leader to be effective. Genuine kindness can be achieved by checking one’s intentions before any interaction and cultivating self-compassion by relinquishing compulsive self-criticism (Hougaard et al., 2020). Furthermore, mindful leaders will use critical reflection to guide their intentions and attention (Annabi, 2021). Through daily practice, mindfulness promotes the mindset of wise compassion, which is the most effective way to lead and support others through difficult times (Hougaard et al., 2020). A mindful, wise, and compassionate leader may play a decisive role in addressing physician burnout, particularly during COVID-19.

### Systems-level culture and learning

To facilitate change, mindful leaders need to become aware of organizational culture and how it influences practice and learning. Schein (2004) defines culture as a pattern of shared principles that a group learns, which is then accepted and taught to new members as the right way to perceive and react to problems. Moreover, organizational culture can be established through coercive persuasion when systems-level beliefs, whether effective or not, are internalized by individuals. Schein (2004) suggests that leaders are tasked with assessing the functionality of the shared assumptions in the deeper levels of a culture as they deal with the anxiety that arises with the introduction of new learning. Individuals, organizations, and systems comfortable in their old ways of practice will naturally resist change, fearing the potential failure of innovation. However, Schein explains that leaders need to motivate followers to unlearn ineffective beliefs and methods while creating a supportive environment that decreases learning anxiety (Coutu, 2002). With new learning, cognitive redefinition occurs, which facilitates the understanding of new concepts and standards for evaluation (Schein, 2004).

Culture change is transformative as it facilitates unlearning old methods of operating and relearning new concepts that can be more effective (Schein, 2004). As they tackle this physician mental health crisis, the task of influencing organizational culture will be the most difficult one faced by leaders aiming to transform institutionalized practices. Otherwise, what awaits will be survival anxiety, which Schein describes as “the horrible realization that in order to make it, you’re going to have to change” (Coutu, 2002, p. 104). Change is inevitable, but with current circumstances surrounding clinician well-being, health systems should not wait for survival anxiety to be the catalyst. A more effective way is to support the physician leaders in reducing the anxiety of learning during a necessary systems-level change to existing culture and practices.

### Limitations and research implications

Despite the specific inclusion criterion that focused on mindfulness training with physicians, there were several limitations in this scoping review. Mindfulness is still a relatively nascent concept, with a paucity of high-quality studies found in the literature. Also, health systems may not be ready to fund research or adapt costly interventions like MBSR. Thus, it comes as no surprise that there were only 31 studies examined for this review.

With 11 RCTs and a small number of participants in the included studies, it is difficult to make generalizable inferences that could instruct current practice. Several studies attest to the low number of participants, the voluntary nature of participation, convenience sampling, and the high likelihood of selection bias affecting the results (Beckman et al., 2012; Kinser et al., 2016; Lebares et al., 2019; Hente et al., 2020). Social desirability bias was also noted as a factor since interventions in many studies were author-led, with reliance on self-report measures (Dobkin et al., 2016; Braun et al., 2019; Ameli et al., 2020). However, Fendel et al. (2020) advise that self-selection is a preferred method with such interventions as mindfulness is more effective with individuals willing to engage with the practice.

The lack of a control group in several NRS was seen to limit the interpretability of findings (Razzaque and Wood, 2016; Bentley et al., 2018; Minichiello et al., 2020). However, even with a ‘life-as-usual’ control group in many RCTs, this was viewed as a disadvantage. In the study by Ireland et al. (2017), the transfer of information between the two groups was challenging to manage, suggesting that the control cohort may have indirectly benefited from the program. Ameli et al. (2020) considered that including an active control group instead would have provided valuable insight into the unique effects of mindfulness for their study. Still, there is a lack of consensus on what represents an ideal control group for mindfulness interventions.

The studies in this scoping review did not evaluate participant demographics and how they may have impacted the outcomes and engagement with mindfulness training. Some studies also included only female participants (Ameli et al., 2020), while others omitted physician personality traits or work culture (Brady et al., 2011). In addition, 10 studies in this scoping review included a mixed group of HCPs such as nurses and support staff. However, several of those studies reported the significance of interprofessional mindfulness, which may offset the professional isolation felt by physicians.

As advised by Munn et al. (2018), scoping reviews highlight gaps in practice and serve as precursors for further research. Health systems and healthcare leadership, for example, were two of the immediately apparent, relevant constructs omitted in the literature included in this scoping review. These omissions could instruct future research on mindfulness practice if they were duly examined. Moreover, oversights within the studies reviewed illustrated a reliance on small samples. Future research should focus on larger sample sizes and consider the use of active control groups. Participant demographics should be assessed, and results correlated with organizational culture and health systems practices. It would also be interesting to study the effects of working full-time or part-time and how the latter may render physicians less susceptible to burnout or more amenable to adhering to daily mindfulness practices. Comparing full-time with part-time physicians may also shed light on current health system culture and traditions and how they may be complicit in perpetuating burnout. Also, research on a heterogeneous group of physicians would allow for comparisons between specialties, while interprofessional programs can potentially foster camaraderie and improve working environments. Specific interventions should then be tailored to staff needs, preferences, and personality traits while also considering the effects of clinical behaviors and how they impact patient care.

This scoping review analyzed the effects of mindfulness on physician burnout. Regarding the research question, the results and emerging themes demonstrated how mindfulness improves physician mental health and well-being. Routine mindfulness practice increases compassion, restores empathy and provides physicians an adjuvant for effective healthcare leadership.

## Conclusion

As a mental health epidemic, burnout poses a serious risk to patients, health systems, and HCPs. This scoping review synthesized current literature on the effects of mindfulness on physician burnout. Results are encouraging but not generalizable. However, mindfulness is a nascent field in research. Despite the scarcity of studies and associated limitations, several themes emerged, which answered the scoping review question and discovered that mindfulness positively impacts physician burnout. These results may also drive future, larger-scale studies. Other determinants of burnout and broader considerations were also discussed, however, the research question was simplistic and initially targeted the extant literature. Further analysis of the studies revealed omissions of healthcare leadership and systems-level influence on physician burnout and mindfulness training, which is the most significant finding in this scoping review.

Mindfulness has shown links to improved perceived well-being and positive psychology. Programs like MBSR and smartphone apps like Headspace are becoming popular methods showing evidence of sustained improvements in mental health. Furthermore, mindfulness practice is inherently linked to effective leadership, strengthened by wise compassion. Like in mindfulness, solutions to complex problems are facilitated by awareness. Health systems must first acknowledge the growing rates of burnout in HCPs. Such significant concerns need to be incorporated into institutional strategy as a priority. Even the most skilled and competent HCP relies on effective mental health and resilience to provide the best possible patient care. As Donald Berwick, a leader in healthcare quality improvement, said: “we cannot relieve the distress of others until we get better at sensing our own, and what we need to relieve it” (Berwick, 2005, p. 125). de Zulueta (2021) attested that leadership is fundamental to healthcare systems capable of providing staff with emotional and practical support. The author affirmed that COVID-19 revealed an inherent weakness in these systems in several ways, not least the policies and procedures designed to mitigate stressors to staff.

Current health systems cannot effectively function during a physician burnout epidemic. Therefore, health organizations should look to their physician leaders for creative solutions. They should explore a systems-level change to the existing organizational culture.

## Author contributions

HM was responsible for the database search, critical analysis, synthesizing themes, and drafting of the manuscript. Both authors contributed to conception and study design, manuscript revision, approving the submitted final manuscript, and agreed to be accountable for all aspects of the work.

## Abbreviations

JD-R: job demands-resource
PCP: primary care physician
HCP: healthcare professional
SIBS: system individual burnout spectrum
PPE: personal protective equipment
PCC: population concept context
NRS: non-randomized studies
SJR: SCImago Journal Rank
ABDC: Australian Business Deans Council
AJG: academic journal guide
MBSR: mindfulness-based stress reduction
MBSC: mindfulness-based self-care
MBCT: mindfulness-based cognitive therapy
MIHP: mindfulness for interdisciplinary health professionals.
